# MALAT1 promotes malignant pleural mesothelioma by sponging miR-141-3p

**DOI:** 10.1515/med-2021-0383

**Published:** 2021-11-03

**Authors:** Pei Wang, Cuiwei Bai, Shasha Shen, Chang Jiang, Jie Deng, Dan Han

**Affiliations:** Department of Medical Imaging, First Affiliated Hospital of Kunming Medical University, Kunming 650032, China; Department of Obstetrics and Gynecology, Luoyang CITIC Central Hospital, Luoyang 471003, China; Department of Medical Imaging, First Affiliated Hospital of Kunming Medical University, No. 295 Xichang Road, Kunming 650032, China

**Keywords:** lncRNA MALAT1, malignant pleural mesothelioma, miR-141-3p, YAP1-Hippo, signaling pathway

## Abstract

The aim of this study was to clarify the role of lncRNA metastasis-associated lung adenocarcinoma transcript 1 (MALAT1) in proliferation, migration, and invasion of malignant pleural mesothelioma (MPM) cells. The quantitative reverse transcription polymerase chain reaction (RT-qPCR) was used to detect the expression of MALAT1 in MPM cell lines. The effects of MALAT1 and miR-141-3p on the proliferation, migration, and invasion of MPM cells were studied through a series of *in vitro* cellular experiments. The flow cytometry was utilized to detect the cell apoptosis. The dual‐luciferase reporter assay was employed to explore the binding relationship among MALAT1, miR-141-3p, and YES-associated protein 1 (YAP1). MALAT1 was overexpressed in MPM cell lines, while its knockdown significantly inhibited the cell proliferation, migration, and invasion, and increased the number of MPM cells in the G0/G1 phase. In addition, MALAT1 could directly bind to miR-141-3p and inhibit its expression. YAP1 has been identified as a downstream target of miR-141-3p, and its expression level was inhibited by miR-141-3p. MALAT1 can be used as a competitive endogenous RNA (ceRNA) to regulate the YAP1-Hippo signaling pathway through miR-141-3p, promote the proliferation, migration, and invasion of MPM cells, and provide a new target for the therapy of MPM.

## 1 Introduction

Malignant pleural mesothelioma (MPM) is a rare and highly aggressive malignant tumor derived from the surface of the pleura, which is associated with a poor prognosis and a median survival time of 8–14 months [1]. It is caused by exposure to asbestos fibers [1]. Besides, it has a long incubation period, and MPM caused by asbestos fibers typically occurs within 20–50 years after exposure. Based on the rate of exposure to asbestos, it is expected that the number of MPM cases will continue to increase in the upcoming decades [2]. MPM patients have no specific primary symptoms, and the main symptoms include dyspnea, chest pain, weight loss, and fatigue [3]. The main therapeutic methods for MPM involve surgery, radiotherapy, and chemotherapy. Single therapy and combination therapy have been extensively studied for MPM, while further therapies should be developed for the advanced stages of the disease [4]. Therefore, it is highly essential to explore the molecular mechanism underlying the occurrence and development of MPM, in order to improve the diagnosis and successful treatment of MPM.

lncRNA metastasis-associated lung adenocarcinoma transcript 1 (MALAT1), also known as nuclear-enriched autosomal transcript 2, is an important member of the lncRNA family. It not only plays a pivotal role in angiogenesis [5], but also influences the occurrence and development of tumors. MALAT1 is abnormally expressed in breast cancer [6], liver cancer [7], gastric cancer [8], pancreatic cancer [9], and other malignant tumors. MALAT1 promotes the proliferation and invasion of tumor cells [10], affects the drug resistance of tumor cells [11], and is associated with the poor prognosis of a variety of solid tumors [12]. At present, there is no report on the cancer-promoting effect of lncRNA MALAT1 on MPM. According to data from The Cancer Genome Atlas (TCGA) database, it was demonstrated that MALAT1 is overexpressed in the majority of epithelioid and biphasic MPM samples [13], confirming that MALAT1 promotes the occurrence and development of MPM.

MicroRNAs (miRNAs) are short (20–24 nt) non-coding RNAs that are involved in post-transcriptional regulation of gene expression in multicellular organisms, and can bind to the 3′-untranslated region (3′-UTR) of target mRNAs. RNA-induced silencing complex is a multi-protein complex, which functions in gene silencing via a variety of pathways at the transcriptional and translational levels [14], thereby inhibiting the expression of the target protein [15]. To date, a large number of studies showed that miRNAs play a significant role in the occurrence, development, invasion, metastasis, proliferation, apoptosis, and drug resistance of various tumors [16]. An evidence demonstrated that the association between lncRNA and miRNA is of great significance to study the occurrence and development of diverse types of cancer [17]. The lncRNA with a targeted binding site can form a competitive endogenous RNA (ceRNA) with miRNA, thereby inhibiting the regulation of the expression of the target gene by the miRNA [18,19].

In the present study, we assessed the overexpression of MALAT1 in MPM cell lines, and confirmed that MALAT1 could form a relationship between ceRNA and miR-141-3p, thereby inhibiting the regulatory effects of miR-141-3p on the target gene (YES-associated protein 1 [YAP1]), leading to dysregulation of the Hippo signaling pathway and promoting the occurrence and development of MPM.

## 2 Methods

### 2.1 Cell lines and cell culture

Normal mesothelial cell line (Met-5A) and MPM cell lines (NCI-H226, NCI-H2452, and MSTO-211H) were purchased from the Cell Bank of Type Culture Collection of the Chinese Academy of Sciences (Shanghai, China). The MPM cell lines were cultured in a Roswell Park Memorial Institute (RPMI)-1640 medium (containing 10% fetal bovine serum [FBS], 100 U/mL penicillin, and 100 mg/mL streptomycin), and the Met-5A was cultured in a M199 medium (in the presence of 10% FBS, 100 U/mL penicillin, and 100 mg/mL streptomycin) in a humid environment at 37°C and 5% CO_2_.

### 2.2 Cell transfection

Lentiviral sh-MALAT1, short hairpin RNA (shRNA) for negative control (sh-NC), lentiviral miR-141-3p mimics, mimics-NC, plasmid si-YAP1, si-NC, miR-141-3p inhibitor, and inhibitor-NC were all synthesized by GenePharma Co., Ltd. (Shanghai, China). Transfection of the cells was undertaken using the Lipofectamine 3000 kit (Thermo Fisher Scientific, Waltham, MA, USA). The transfection efficiency was assessed by the quantitative reverse transcription polymerase chain reaction (RT-qPCR).

### 2.3 RT-qPCR

TRIzol reagent (TaKaRa, Tokyo, Japan) was used to extract total RNA from Met-5A and MPM cell lines. The NanoDrop was utilized to detect the concentration and quality of RNA. We also employed the PrimeScript RT Master Mix kit (TaKaRa, Tokyo, Japan) with gDNA Eraser and Mir-X miRNA RT-qPCR SYBR kit (TaKaRa, Tokyo, Japan) to convert the extracted RNA into cDNA. The cDNA template was mixed with gene-specific primers and SYBR Green PCR Master Mix (Roche, Basel, Switzerland). The RT-qPCR was performed on TaqMan®Universal PCR Master Mix II (Applied Biosystems, Waltham, MA, USA). All primers were purchased from Invitrogen (Carlsbad, CA, USA), and the sequences of primers used for RT-qPCR are listed in Table 1. Glyceraldehyde 3-phosphate dehydrogenase (GAPDH) and U6 were used as internal controls. The 2^−ΔΔCt^ method was used to calculate the relative expression level.

**Table 1:** Sequences of primers used for RT-qPCR

Gene	Forward primer sequence	Reverse primer sequence
lncRNA MALAT1	AACTGGGGGTTGGTCTGG	AAATTCCAAAAGAGAACCACACA
Hsa-miR-141-3p	ACACTGTCTGGTAAAGATGG	mRQ 3′ Primer (Cat. Nos. 638313, Takara Bio USA, Inc.)
YAP1	TGAACAAACGTCCAGCAAGATAC	CAGCCCCCAAAATGAACAGTAC
TEAD1	GAAAACATGGAAAGGATGAGTG	GGCTATCAATTCATTCCTACC
GAPDH	AATCCCATCACCATCTTCCA	TGGACTCCACGACGTACTCA
U6	CTCGCTTCGGCAGCACA	AACGCTTCACGAATTTGCGT

### 2.4 Cell counting kit-8 (CCK-8) assay

The CCK-8 assay was carried out to detect the cell proliferation ability. MPM cells (5 × 10^3^ cells/well) were seeded into 96-well plates, each experiment was repeated 3 times, and then were incubated for 24, 48, and 72 h. Subsequently, 20 μL of CCK-8 solution (Dojindo Molecular Technologies, Inc., Rockville, MD, USA) was added to each well and incubated at 37°C for 2 h. The absorbance was measured at 450 nm using a microplate reader (Thermo Fisher Scientific, Waltham, MA, USA). The cell growth curve was drawn with time as the horizontal axis and optical density value as the vertical axis. The experiment was repeated three times.

### 2.5 Flow cytometry

The BD FACSCelesta Flow Cytometer (BD Biosciences, San Jose, CA, USA) was used to analyze the percentage of cells in different phases of the cell cycle. Discard the original culture medium, wash the cells twice with phosphate-buffered saline (PBS), trypsinize, add culture medium to stop the digestion, discard the supernatant after centrifugation, and add a small amount of PBS to keep it moist. Add 25 μL of 5 μg/mL 4′,6-diamidino-2-phenylindole solution to each tube containing 0.5 mL of cultured cells, and let stand on ice for 30 min prior to data acquisition. Collect data and use CytExpert 2.3 software (Beckman Coulter Inc., Brea, CA, USA) for analysis.

The Annexin V-FITC Apoptosis Detection kit (Dojindo Molecular Technologies, Inc., Rockville, MD, USA) was used to analyze the apoptosis of the cells. First, collect the culture medium of the cells, wash the adherent cells once with PBS, eluate and collect the cells with TrypLE TM Express (Gibco, NY, USA), gently resuspend the cells in PBS, and count them. Incubate the solution in dark condition for 20 min at room temperature. Finally, the BD FACSCelesta Flow Cytometer was used to detect the cell apoptosis, and the data were analyzed by the FlowJo 11.0 software (Tree Star, Inc., San Carlos, CA, USA) to determine the rate of apoptosis.

### 2.6 EdU assay

The EdU Cell Proliferation kit (Beyotime, Shanghai, China) was used to evaluate the proliferation ability of the transfected cells. The cells were treated with EdU working solution for 2 h. According to the manufacturer’s instructions, fix the cells with 4% paraformaldehyde at room temperature, treat with penetrant, stain the cells with EdU staining kit (Beyotime, Shanghai, China), and visualize using an inverted fluorescence microscope (Olympus, Tokyo, Japan) to assess the proliferation of the cells.

### 2.7 Transwell invasion assay

The cells were cultured for 24 h without serum in advance and then were digested with trypsin and counted. Next 3 × 10^4^ cells were inoculated into the upper compartment of the Transwell chamber (Corning Inc., Corning, NY, USA), and the cells were suspended in a 200 μL medium without serum. In order to perform the Transwell invasion assay, the membrane of the upper compartment was pre-coated with Matrigel, a 500 μL medium containing 10% FBS was added into the lower compartment and then the cells were placed in an incubator for 24 h. Afterward, the cells were washed with PBS, fixed with methanol, and stained with crystal violet. Five fields of view were randomly selected under an upright microscope (Leica, Wetzlar, Germany) to investigate the cell invasion ability. The ImageJ software was utilized to count the invaded cells.

### 2.8 Colony formation assay

After the elution of cells with trypsin, the cells were seeded into a 6-well plate at a density of 500 cells/well, and were then cultured under normal conditions in a RPMI-1640 medium containing 10% FBS (Gibco, NY, USA) for 14 days. In order to visualize and count the colonies, methanol and 0.5% crystal violet (Sigma-Aldrich, USA) were used to fix and stain the colonies, respectively.

### 2.9 Scratch assay

The scratch assay was carried out to assess the migration ability of the cells. First, use a marker to draw two parallel lines on the back of the 6-well plate and then inoculate the cells into the 6-well plate. When the cell growth completely covered the bottom of the plate, use a 10 μL pipette tip to gently draw a line on the plate. After rinsing the cells with PBS, they were visualized at 0, 6, 12, and 24 h after incubation using an inverted microscope.

### 2.10 Western blot assay

First, wash the cells with PBS and then lyse the cells or tissues with phenylmethylsulfonyl fluoride-containing RIPA lysis buffer (Beyotime Shanghai, China) at 4°C for 1 h. After that, the cells were mixed with 5× sodium dodecyl sulfate (SDS) loading buffer and boiled at 100°C for 5 min. The sample was centrifuged at 12,000 rpm for 25 min at 4°C, and the supernatant was then collected. The proteins (70 mg of each extract) were separated by 12% SDS-polyacrylamide gel electrophoresis, electroblotted onto polyvinylidene fluoride membranes, and the membranes were blocked with 5% skimmed milk at room temperature. The membranes were incubated with anti-YAP1 antibody (dilution, 1:500; catalog no. ab52771; Abcam, Cambridge, UK) at 4°C overnight. After rinsing thrice with Tris-Hcl+Tween20 (TBST) for 15 min, add horseradish peroxidase-labeled goat anti-rabbit secondary antibody (dilution, 1:5,000; catalog no. ab205718; Abcam) to the membranes, incubate for 1 h, and wash twice with TBST for 10 min. The enhanced chemiluminescence detection system (Thermo Fisher Scientific, USA) was used to visualize immunoreactive proteins, and the imaging system (Bio-Rad Laboratories Inc., Hercules, CA, USA) was employed to analyze the protein expression. The relative level of protein was regarded as the ratio of the gray value of target band to that of the internal reference band, with GAPDH serving as an internal reference.

### 2.11 Dual-luciferase reporter assay

The TargetScan 7.2 (http://www.targetscan.org/vert_72) and starBase 2.0 (http://starbase.sysu.edu.cn) were, respectively, used to predict the potential of hsa-miR-141-3p and lncRNA MALAT1 target. The wild-type (WT) or mutant (MUT) 3′-UTR sequence of MALAT1 and YAP1 was cloned into the pmirGLO luciferase reporter vector (Promega, Madison, WI, USA). NCI-H226 cells (3 × 10^4^ cells/well) were seeded into a 24-well plate and combined with miR-141-3p mimics, NC-mimics, pmirGLO-WT-MALAT1, pmirGLO-MUT-MALAT1, pmirGLO-WT-YAP1, and pmirGLO-MUT-YAP1. After 48 h of transfection, the Dual-Luciferase Reporter Assay system (Promega, Madison, WI, USA) was utilized to measure the luciferase activity.

### 2.12 Tumor xenograft model

Female BALB/C nude mice were grown under specific pathogen-free conditions for 4–6 weeks, and were randomly divided into two groups (*n* = 6 mice in each group). The cultured sh-NC and sh-MALAT1-transduced NCI-H226 cells were eluted with trypsin and counted, and 1 × 10^6^ cells/100 μL were subcutaneously transplanted into the right axilla of each nude mouse. The tumor growth was monitored on days 1, 5, 10, 15, 20, 25, and 30, and the tumor size was measured and calculated as follows: *V* = (*L* × *W*2)/2, where *V* is tumor volume, *W* is tumor width, and *L* is tumor length. All animals were sacrificed, and the transplanted tumor tissue was excised for further research. This study was approved by the Ethics Committee of Kunming Medical University (Approval No. Kmmu2021726).

### 2.13 Statistical analysis

The statistical analysis was performed using the SPSS 22.0 software, and the data were expressed as the mean value ± standard deviation (SD). The independent-samples *t*-test and one-way analysis of variance (ANOVA) were employed to analyze data and compare differences. All experiments were repeated at least three times, and *P* < 0.05 was regarded statistically significant. The GraphPad Prism 8.0 (GraphPad Software Inc., San Diego, USA) was utilized to draw the curves.

## 3 Results

### 3.1 lncRNA MALAT1 was highly expressed in MPM cell lines

**Figure 1:**
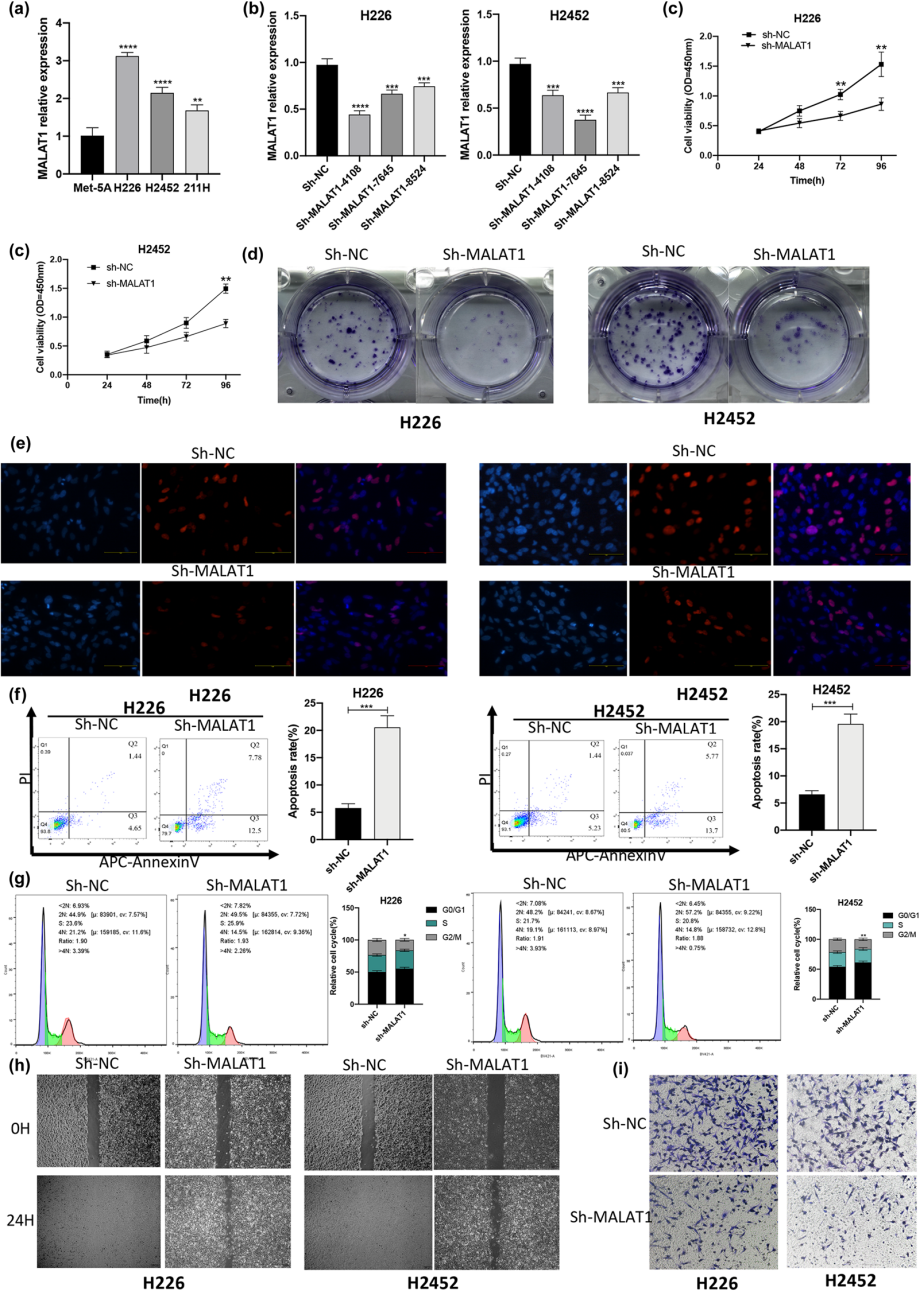
lncRNA MALAT1 is overexpressed in MPM cell lines. Knockdown of MALAT1 inhibits the proliferation, migration, and invasion of MPM cells, and induces the cell apoptosis. (a) The expression level of MALAT1 in human MPM cell lines (H226, H2452, and MSTO-211H) and normal human epithelial cell lines (Met-5A) was detected by RT-qPCR (**P < 0.01, ****P < 0.0001 vs Met-5A group, one-way ANOVA and SNK-Q tests). (b) The knockdown efficiency of sh-MALAT1#4108, sh-MALAT1#7645, and sh-MALAT1#8524 in H226 and H2452 cells was assessed by RT-qPCR (***P < 0.001, ****P < 0.0001 vs Sh-NC group, one-way ANOVA and SNK-Q tests). (c) The influence of sh-MALAT1 on the cell viability was detected by CCK-8 (**P < 0.01, *t*-test). (d) The effect of sh-MALAT1 on the cell proliferation was evaluated by colony formation assay. (e) The effect of sh-MALAT1 on the cell proliferation was assessed by EdU assay. (f) The effect of sh-MALAT1 on the cell apoptosis was determined by flow cytometry (***P < 0.001, *t*-test). (g) Flow cytometry detected the influence of sh-MALAT1 on the cell cycle progression (*P < 0.05, **P < 0.01, chi-square test). (h) The effect of sh-MALAT1 on the cell migration was determined by scratch test. (i) The effect of sh-MALAT1 on the cell invasion was investigated by Transwell invasion assay. RT-qPCR, quantitative reverse transcription polymerase chain reaction; NC, negative control; sh-RNA, short hairpin-RNA.

The RT-qPCR was used to detect the expression level of MALAT1 in different MPM cell lines (NCI-H226, NCI-H2452, and MSTO-211H) and in Met-5A cell line. The results showed that the expression level of MALAT1 was significantly elevated in these MPM cell lines, especially in NCI-H226 and NCI-H2452 (Figure 1a). Therefore, NCI-H226 and NCI-H2452 cell lines were selected for the next experiments.

### 3.2 Knockdown of MALAT1 inhibited the proliferation, migration, and invasion of MPM cells, and induced the cell apoptosis

The NCI-H226 and NCI-H2452 cells were transfected with shRNA-negative control (shRNA-NC) and MALAT1-shRNA (shRNA4108, shRNA7645, and shRNA8524). Compared with the sh-NC group, the expression level of MALAT1 was markedly reduced in the sh-MALAT1 group, especially in the H226-shRNA4108 and H2452-shRNA7645 groups (Figure 1b). Therefore, the H226-shRNA4108 and H2452-shRNA7645 groups were selected for the subsequent experiments.

In order to explore the relationship between the expression level of MALAT1 and proliferation, migration, invasion, and apoptosis of MPM cells, shRNA-MALAT1 (sh-MALAT1) and sh-NC were transfected into two MPM cell lines: NCI-H226 and NCI -H2452. The cell proliferation ability was tested by the CCK-8, colony formation, and EdU assays. The results of the CCK-8 assay showed that compared with the sh-NC group, the cell proliferation ability of the sh-MALAT1 group was reduced (Figure 1c). The results of the colony formation revealed that compared with the sh-NC group, the colony formation ability of the sh-MALAT1-H226 and sh-MALAT1-H2452 groups was significantly reduced (Figure 1d). The results of the EdU assay indicated that compared with the sh-NC group, the proliferation of sh-MALAT1-H226 and sh-MALAT1-H2452 cells was significantly attenuated (Figure 1e). According to the findings attained by the flow cytometry, compared with the sh-NC group, the number of apoptotic cells in the cells transfected with sh-MALAT1 was elevated (Figure 1f), and knockdown of MALAT1 increased the proportion of MPM cells in the G0/G1 phase (Figure 1g).

The migration and invasion abilities of the MPM cell lines were tested by scratch test and Transwell invasion assay, respectively. The results of scratch test showed that knockdown of MALAT1 reduced the migration ability of H226 and H2452 cells (Figure 1h). According to the results of Transwell invasion analysis, compared with the sh-NC group, the invasion ability of the sh-MALAT1-H226 and sh-MALAT1-H2452 groups was significantly reduced (Figure 1i). Collectively, the si-MALAT1 could noticeably downregulate the expression level of MALAT1, while MALAT1 could reduce the proportion of MPM cells in the G0/G1 phase, inhibit the cell apoptosis, and promote the proliferation, migration, and invasion abilities of MPM cells.

### 3.3 Knockdown of MALAT1 inhibited the growth of MPM cells *in vivo*

**Figure 2:**
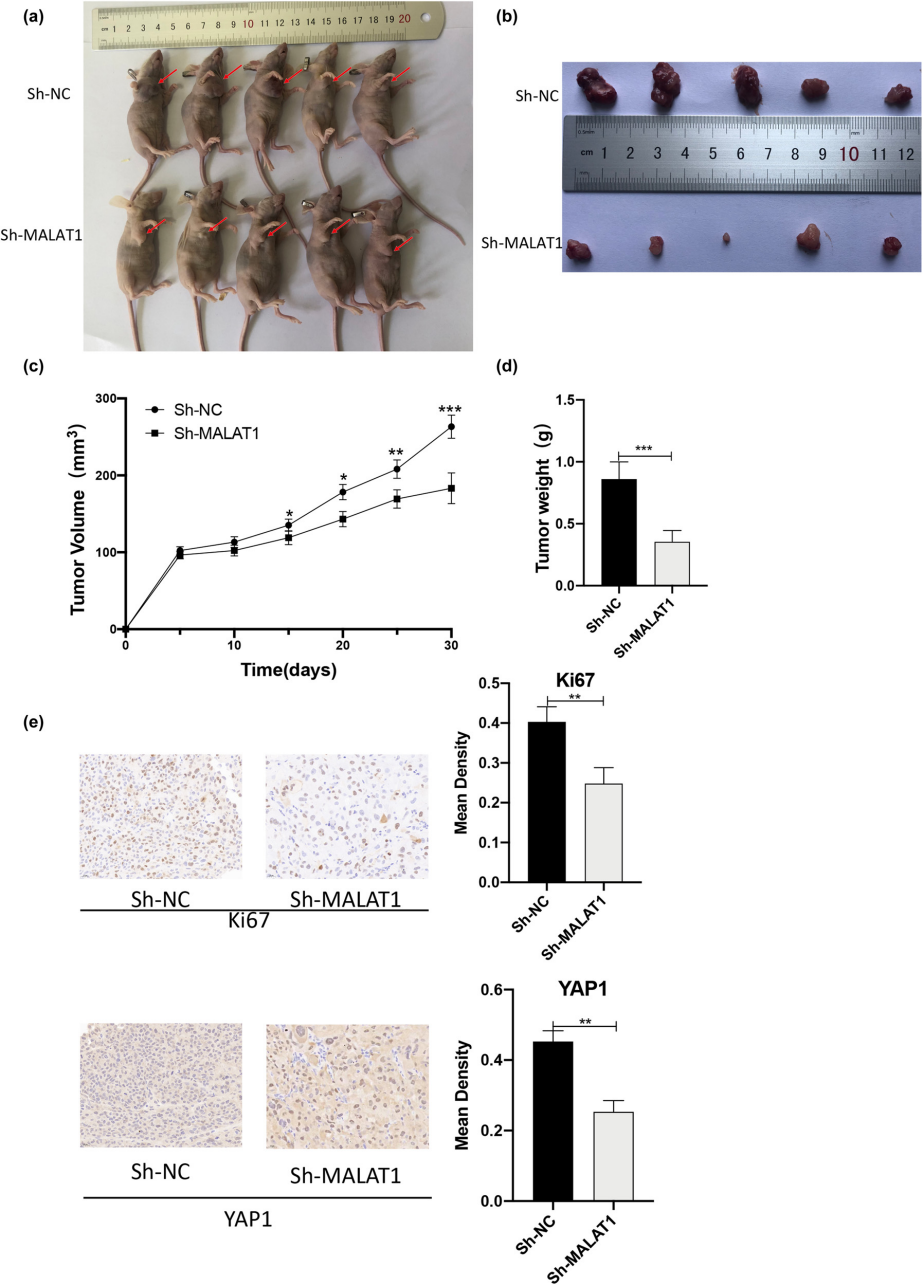
Knockdown of MALAT1 inhibits the growth of MPM cells *in vivo*. (a and b) sh-MALAT1 inhibits the growth of MPM cells in xenograft mice. It can be seen that the subcutaneous transplanted tumor is located in the right axilla of nude mice, and the volume of Sh-MALAT1 group is significantly smaller than that of Sh-NC group. (c and d) sh-MALAT1 inhibits volume and weight of MPM tumor (*n* = 5 per group, *P < 0.05, **P < 0.01, ***P < 0.001, *t*-test). (e) IHC staining showed that the expressions of Ki67 and YAP1 were reduced in xenograft tumor tissues of mice that received sh-MALAT1-transfected H226 cells (**P < 0.01, *t*-test).

The effects of MALAT1 on the growth of MPM cells were examined *in vivo*. Five weeks after transplantation, tumors were collected and cut into small fragments. Compared with the sh-NC group, the nude mice in the sh-MALAT1 group had significantly smaller tumors (Figure 2a and b). Compared with the sh-NC, the growth rate and weight of tumors in the sh-MALAT1 group were also significantly reduced (Figure 2c and d). Immunohistochemical (IHC) staining showed that the expression levels of Ki67 and YAP1 in xenograft tumor tissues of nude mice injected with sh-MALAT1-transfected cells were markedly downregulated (Figure 2e).

### 3.4 lncRNA MALAT1 directly targeted miR-141-3p and reduced its level

To our knowledge, lncRNA can act as a miRNA sponge and inhibit the activity of miRNA. However, it is essential to indicate whether MALAT1 can regulate miRNA level in a spongy form in MPM cells?

First, the starBase (ver. 2.0) was utilized to search for miRNAs with complementary base pairings with MALAT1. Next we concentrated on miR-141-3p, which is a tumor suppressor involved in proliferation, migration, and invasion of cancer cells.

**Figure 3:**
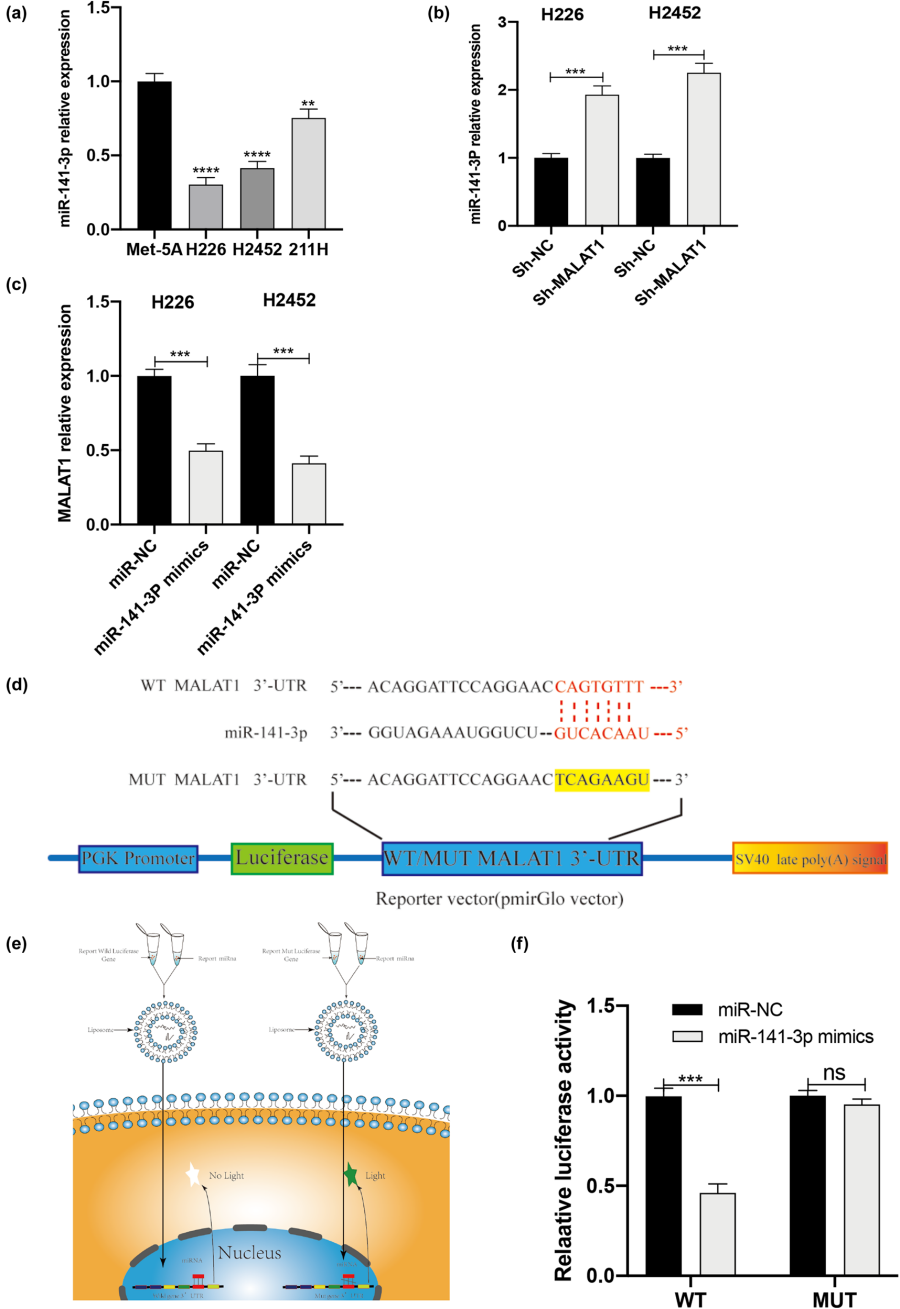
MALAT1 directly binds to the expression level of miR-141-3p and reduces its expression level. (a) The expression level of miR-141-3p in human MPM cell lines (H226, H2452, and MSTO-211H) and normal human epithelial cell lines (Met-5A) was detected by RT-qPCR (**P < 0.01, ****P < 0.0001 vs Met-5A group, one-way ANOVA and SNK-Q tests). (b) The RT-qPCR showed that the expression of miR-141-3p in the sh-MALAT1 group was elevated compared with that in the sh-NC group (***P < 0.001, *t*-test). (c) The RT-qPCR indicated that the expression of MALAT1 in the miR-141-3p mimics group was reduced compared with that in the miR-NC group (***P < 0.001, *t*-test). (d) The miR-141-3p target binding site was predicted in MALAT1. (e) This image illustrates how the luciferase assay could measure the binding. (f) The effect of miR-141-3p mimics on the luciferase activity of reporter genes with WT or MUT MALAT1 target regions (***P < 0.001, *t*-test).

The miR-141-3p level in MPM cells was detected by the RT-qPCR. The miR-141-3p level in NCI-H226, NCI-H2452, and MSTO-211H cells was reduced compared with that in Met-5A cells (Figure 3a). The RT-qPCR revealed that the miR-141-3p level was elevated in the sh-MALAT1 group compared with that in the sh-NC group (Figure 3b); besides, compared with the control group (miR-NC), the MALAT1 level in the miR-141-3p mimics group was reduced (Figure 3c). Consequently, the levels of miR-141-3p and MALAT1 were negatively correlated together in MPM cells.

Furthermore, the dual-luciferase reporter assay was used to explore the targeted binding relationship between miR-141-3p and MALAT1. For this purpose, the StarBase (ver. 2.0) was employed to determine the complementary binding site between miR-141-3p and 3′-UTR of MALAT1 (Figure 3d). We cloned the predicted miR-141-3p binding site (MALAT1-WT) and the mutant binding site (MALAT1-MUT) of MALAT1 into the luciferase reporter plasmids. The luciferase activity was measured 24 h after transient co-transfection of plasmids. The results also indicated that, compared with mimics-NC, miR-141-3p mimics significantly reduced the luciferase activity of MALAT1-WT. Additionally, the co-transfection of miR-141-3p mimics and MALAT1-MUT did not change the luciferase activity (Figure 3f). Taken together, MALAT1 could directly bind to miR-141-3p and reduce its level.

### 3.5 Overexpression of miR-141-3p inhibited the proliferation, migration, and invasion of MPM cells, and induced the cell apoptosis

**Figure 4:**
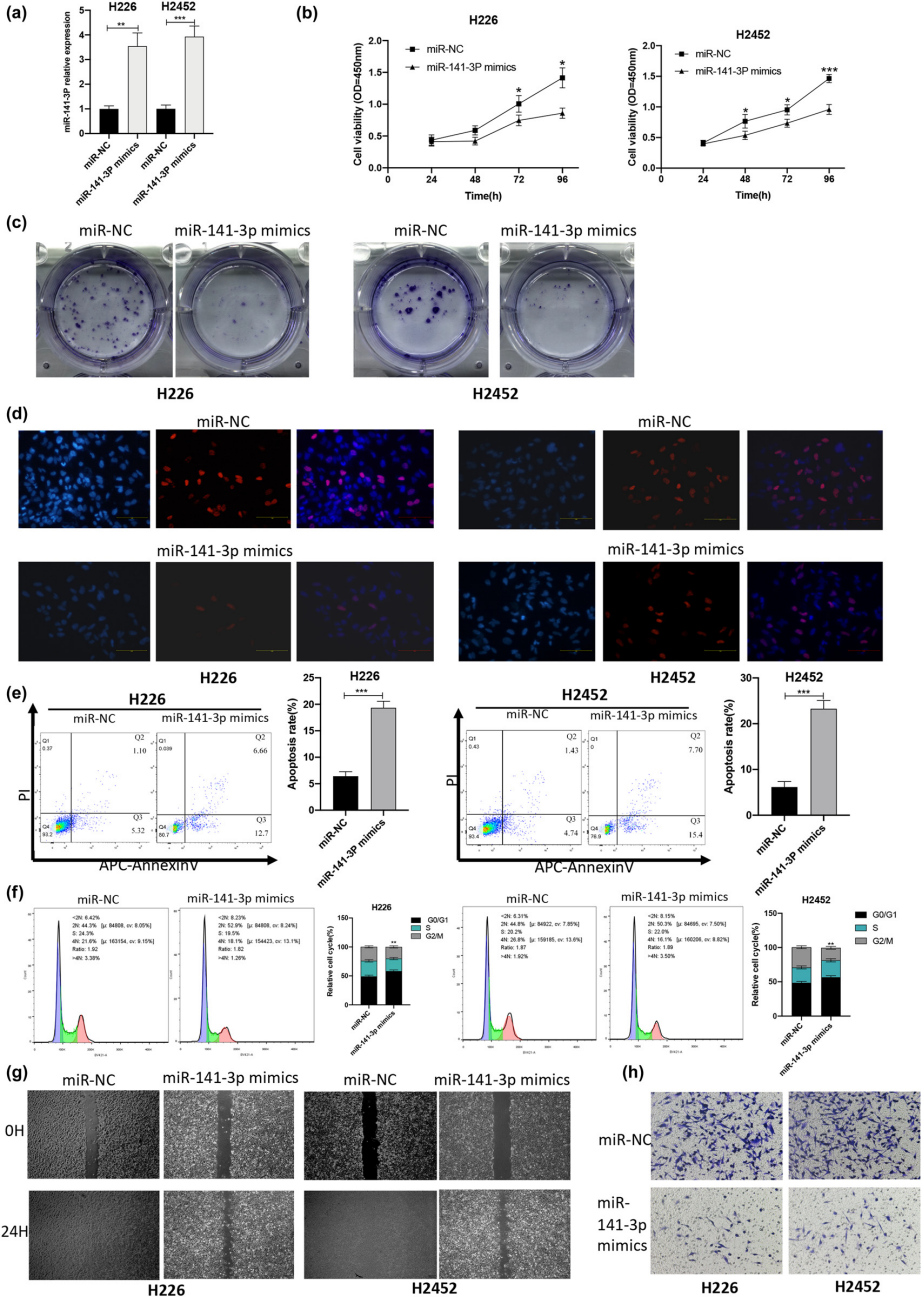
Overexpression of miR-141-3p inhibits proliferation, migration, and invasion of MPM cells, and induces the cell apoptosis. (a) The RT-qPCR showed that the expression of miR-141-3p in the miR-141-3p mimics group was higher than that in the miR-NC group (**P < 0.01, ***P < 0.001, *t*-test). (b) The effect of miR-141-3p mimics on the cell viability was detected by CCK-8 (*P < 0.05, ***P < 0.001, *t*-test). (c) The influence of miR-141-3p mimics on the cell proliferation was assessed by colony formation assay. (d) The effect of miR-141-3p mimics on the cell proliferation was evaluated by EdU assay. (e) The influence of miR-141-3p mimics on the cell apoptosis was determined by flow cytometry (***P < 0.001, *t*-test). (f) Flow cytometry detected the effect of miR-141-3p mimics on the cell cycle progression (**P < 0.01, chi-square test). (g) The influence of miR-141-3p mimics on the cell migration was investigated by scratch test. (h) The effect of miR-141-3p mimics on the cell invasion was detected by Transwell invasion assay.

The RT-qPCR revealed that the miR-141-3p level in the miR-141-3p mimics group was higher than that in the miR-NC group (Figure 4a). The results of CCK-8 assay showed that, compared with the miR-NC group, the cell proliferation ability of the miR-141-3p mimics group was reduced (Figure 4b). The results of the colony formation assay showed that compared with the miR-NC group, the colony formation ability of the miR-141-3p mimics group was significantly attenuated (Figure 4c). The EdU assay indicated that compared with the miR-NC group, the proliferation ability of the miR-141-3p mimics group was significantly declined (Figure 4d). According to the results of flow cytometry, compared with the miR-NC group, the number of apoptotic cells in the miR-141-3p mimics group was elevated (Figure 4e), while overexpression of miR-141-3p increased the proportion of MPM cell lines in the G0/G1 phase (Figure 4f).

The results of scratch test showed that overexpression of miR-141-3p reduced the migration ability of H226 and H2452 cells (Figure 4g). On the basis of the results of Transwell invasion assay, compared with the miR-NC group, the invasion ability of H226 cells in the miR-141-3p mimics and that of H2452 cells in the miR-141-3p mimics were significantly reduced (Figure 4h). The abovementioned findings indicated that overexpression of miR-141-3p could inhibit the proliferation, migration, and invasion of MPM cells, and also induce the cell apoptosis.

### 3.6 MALAT1 upregulated the expression of YAP1 by targeting miR-141-3p

**Figure 5:**
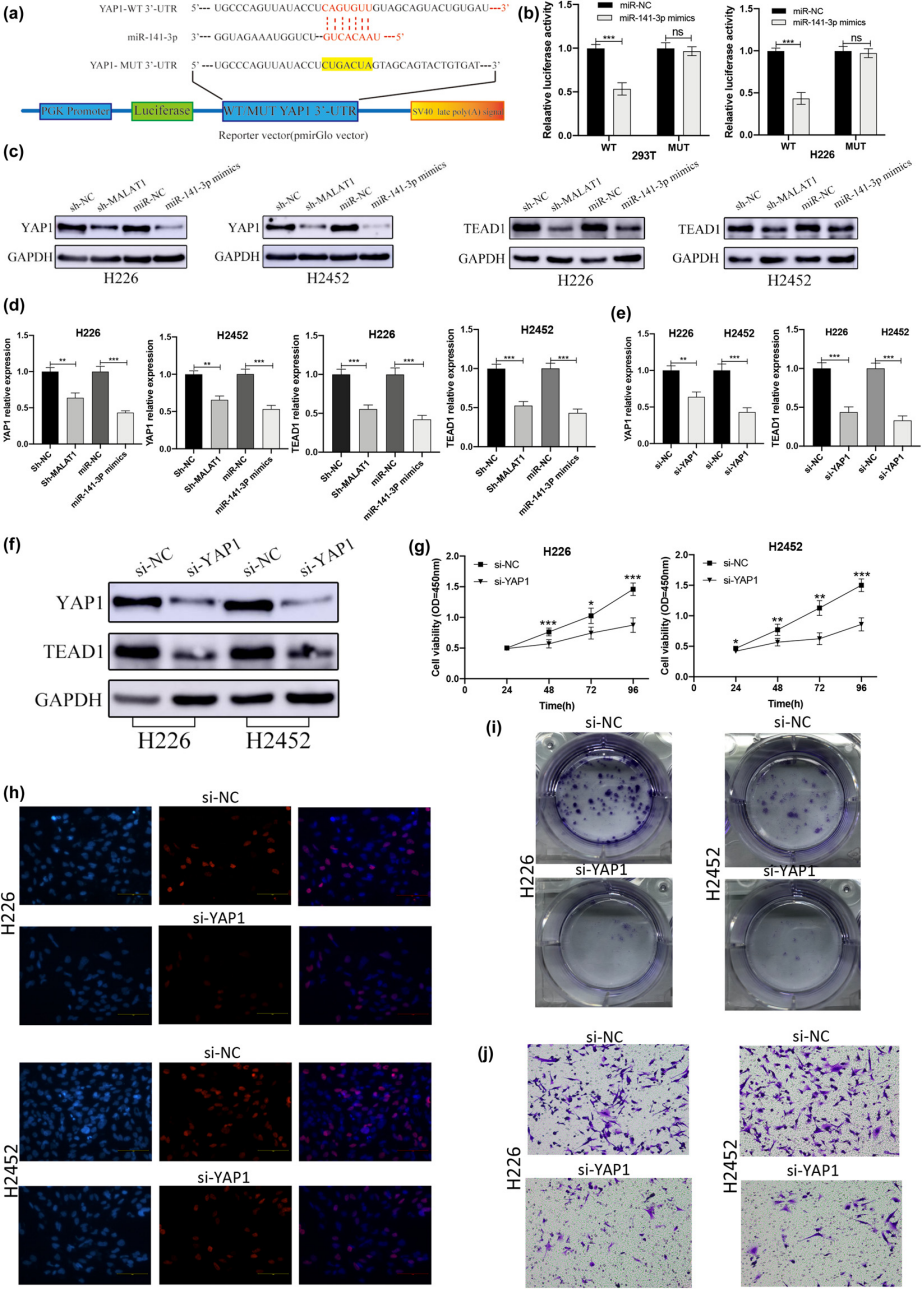
YAP1 is the direct target of miR-141-3p, and knockdown of YAP1 inhibits the proliferation of MPM cells. (a) Prediction of the binding site between miR141-3p and YAP1. (b) The effect of miR-141-3p mimics on the luciferase activity of genes with WT or MUT YAP1 target regions (***P < 0.001, *t*-test). (c) The effects of sh-MALAT1 and miR-141-3p mimics on the expression levels of YAP1 and TEAD1 proteins were detected by Western blotting. (d) The influences of sh-MALAT1 and miR-141-3p mimics on the mRNA expression levels of YAP1 and TEAD1 were detected by the RT-qPCR (**P < 0.01, ***P < 0.001, *t*-test). (e) The RT-qPCR showed that the mRNA expression levels of YAP1 and TEAD1 in si-YAP1-transfected cells were lower than those in si-NC group (**P < 0.01, ***P < 0.001, *t*-test). (f) Western blotting showed that the expression levels of YAP1 and TEAD1 in si-YAP1 transfected cells were lower than those in the si-NC group. (g) The effect of si-YAP1 on the cell viability was determined by CCK-8 (*P < 0.05, **P < 0.01, ***P < 0.001, *t*-test). (h) The effect of si-YAP1 on the cell proliferation was detected by EdU assay. (i) The effect of YAP1 on the cell proliferation was evaluated using the colony formation assay. (j) The effect of YAP1 on the cell invasion was detected by Transwell invasion assay.

We analyzed the regulatory relationship between the levels of miR-141-3p and YAP1. First, the TargetScan 7.2 was employed to predict the binding site between miR141-3p and YAP1 (Figure 5a). Then, the targeted binding relationship between miR-141-3p and YAP1 was explored using the dual-luciferase reporter assay. We cloned YAP1-predicted miR-141-3p binding site (YAP1-WT) and a mutated binding site (YAP1-MUT) into the luciferase reporter plasmid. The luciferase activity was measured 24 h after transient co-transfection. The results showed that compared with the mimics-NC, miR-141-3p mimics significantly reduced the luciferase activity of YAP1-WT, while YAP1-MUT remained unchanged (Figure 5b).

In order to determine the regulatory relationship among MALAT1, miR-141-3p, and YAP1, the effects of miR-141-3p and MALAT1 on YAP1 mRNA/protein expression in human MPM cells were assessed. The results of Western blotting confirmed that both knockdown of MALAT1 and overexpression of miR-141-3p downregulated the expression level of YAP1 and TEA domain family member 1 (TEAD1) (Figure 5c). Similar results were also found at the mRNA level (Figure 5d). Besides, the effects of the expression level of YAP1 and TEAD1 on MPM cells were evaluated. The RT-qPCR showed that compared with the si-NC group, si-YAP1 significantly reduced the expression of YAP1 and TEAD1 in NCI-H226 and NCI-H2452 cells (Figure 5e). Western blotting indicated that compared with the si-NC group, si-YAP1 significantly reduced the expression level of YAP1 and TEAD1 in NCI-H226 and NCI-H2452 cells (Figure 5f). The results of CCK-8 (Figure 5g), EdU (Figure 5h), and colony formation (Figure 5i) assays showed that the inhibition of YAP1 by si-YAP1 decreased the proliferation ability of MPM cells.

According to the results of Transwell invasion analysis, compared with the si-NC group, the invasion ability of the si-YAP1-H226 and si-YAP1-H2452 groups was significantly reduced (Figure 5j). Collectively, YAP1 could promote the proliferation of MPM cells, and MALAT1 interacted with miR-141-3p to regulate the progression of MPM cells by targeting YAP1.

### 3.7 MALAT1 promoted MPM through the YAP1-TEAD1-Hippo signaling pathway mediated by miR-141-3p

**Figure 6:**
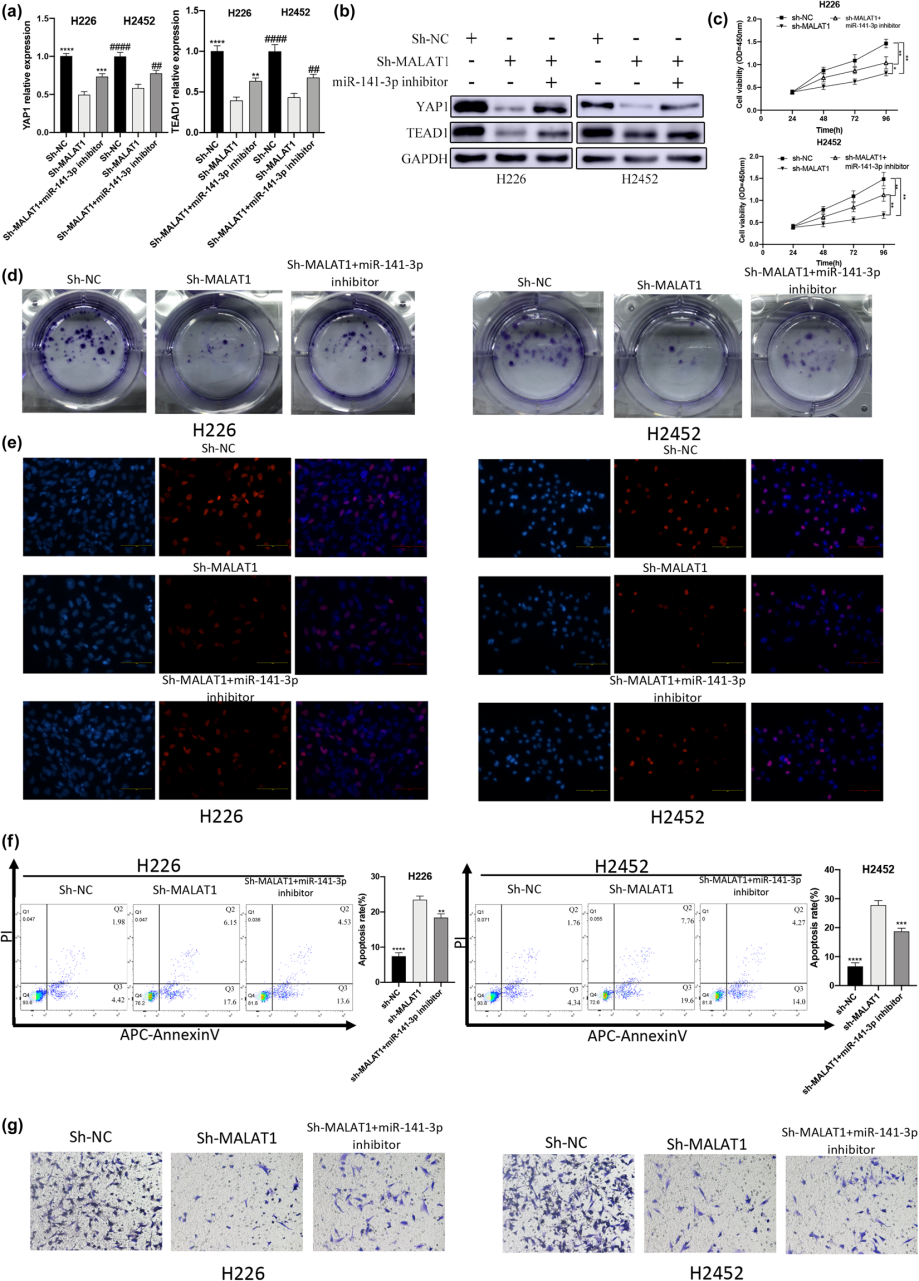
MALAT1 promotes the development of MPM through the YAP1-Hippo signaling pathway mediated by miR-141-3p. (a) The RT-qPCR showed that the decrease in mRNA levels of YAP1 and TEAD1 caused by knockdown of MALAT1 can be rescued by miR-141-3p inhibitor (**P < 0.01, ***P < 0.001, ****P < 0.0001 vs Sh-MALAT1 [H226] group; ^##^ *P* < 0.01, ^####^ *P* < 0.0001 vs Sh-MALAT1 [H2452] group, one-way ANOVA and SNK-Q tests). (b) Western blotting revealed that the decrease in the expression levels of YAP1 and TEAD1 caused by knockdown of MALAT1 can be rescued by miR-141-3p inhibitor. (c) CCK-8 was used to detect the effects of sh-MALAT1 and miR-141-3p on the cell viability (*P < 0.05, **P < 0.01, *t*-test). (d) The influences of sh-MALAT1 and miR-141-3p on the cell proliferation were assessed by colony formation assay. (e) The effects of sh-MALAT1 and miR-141-3p on the cell proliferation were detected by EdU assay. (f) The influences of sh-MALAT1 and miR-141-3p on the cell apoptosis were determined by flow cytometry (**P < 0.01, ***P < 0.001, ****P < 0.0001 vs Sh-MALAT1 group, one-way ANOVA and SNK-Q tests). (g) The effects of sh-MALAT1 and miR-141-3p on the cell invasion were determined by Transwell invasion assay.

In order to determine the biological function of the miR-141-3p/YAP1-TEAD1-Hippo signaling pathway in the progression of MPM cells, a rescue experiment was designed and tested on H226 and H2452 cells. The results of RT-qPCR showed that the decrease in YAP1 and TEAD1 mRNA levels caused by knockdown of MALAT1 was rescued by the miR-141-3p inhibitor (Figure 6a). Similar results were found at the protein level (Figure 6b). According to the results of CCK-8 (Figure 6c), colony formation (Figure 6d), and EdU (Figure 6e) assays, transfection with miR-141-3p inhibitor could rescue sh-MALAT1, causing a decrease in the cell proliferation ability. The increase in the cell apoptosis caused by sh-MALAT1 could also be rescued by miR-141-3p inhibitor (Figure 6f). Additionally, the decrease in invasion ability by sh-MALAT1 could be rescued by miR-141-3p inhibitor (Figure 6g). Consequently, MALAT1 acted as a ceRNA to regulate MPM through the miR-141-3p/YAP1-TEAD1-Hippo signaling pathway.

## 4 Discussion

MPM is a rare and aggressive malignancy arising from the mesothelial cells lining the pleural cavity. Early diagnosis of MPM is primarily compromised by the extremely long latency period pertaining to the development of the tumor. Radiological features, such as unilateral pleural effusion, nodular thickening of pleura, and interlobar fissure thickening are suggestive of MPM. A large number of previous studies demonstrated that the abnormal expression of MALAT1 is related to tumor proliferation and metastasis, indicating that the increased expression of MALAT1 may be a valuable predictive biomarker for the poor prognosis of different types of cancer. For instance, the high expression of MALAT1 is positively correlated with tumor size and lymph node metastasis in patients with non-small cell lung cancer, while is negatively associated with overall survival [20]. In addition, after transducing H226 and H2452 cells with sh-MALAT1, we, in the present research, noticed that sh-MALAT1 significantly inhibited the proliferation of MPM cells, induced the cell cycle arrest in the G1/S phase, and promoted the apoptosis of MPM cells *in vitro*. Additionally, *in vivo* experiments partially confirmed the abovementioned findings, in which knockdown of MALAT1 is highly associated with the size and growth rate of tumors in nude mice. Thus, MALAT1 is an oncogene of MPM, and inhibition of MALAT1 may be a promising treatment for MPM patients.

The miR-200 family has five members, including miR-200a, miR-200b, miR-200c, miR-141, and miR-429 [21]. The miR-200 family gene is considered as a tumor suppressor gene, and its abnormal expression is correlated to the occurrence and development of diverse types of cancer, and also participates in epithelial-mesenchymal transition (EMT) in cancer [22]. The miR-200 family is abnormally expressed in a variety of malignant tumors, such as ovarian cancer [23], pancreatic cancer [24], and colorectal cancer [25]. Li et al. reported [26] that miR-141-3p promotes the proliferation of prostate cancer cells by inhibiting the expression of KIF9. Wang et al. demonstrated [27] that miR-141-3p, as a tumor suppressor gene in glioma cells, downregulates the expression of ATF5, inhibits the proliferation of glioma cells, and promotes the cell apoptosis. Recent data indicated that lncRNA, acting as a natural miRNA sponge, can epigenetically regulate the gene expression by competing for endogenous miRNA binding, thereby reducing the binding of miRNA to target genes [28]. In order to explore whether there is a similar correlation between MALAT1 and miR-141-3p, we conducted bioinformatics analysis and the results proved the putative binding site of miR-141-3p in MALAT1. The results of dual-luciferase reporter assay showed that miR-141-3p can directly bind to MALAT1 through the putative miRNA response element. In addition, knockdown of MALAT1 resulted in the increased expression of miR-141-3p, while overexpression of miR-141-3p suppressed the expression of MALAT1. The abovementioned results indicated a negative regulatory relationship between MALAT1 and miR-141-3p. In addition, miR-141-3p inhibitors exhibited to rescue the tumor suppressive effects of sh-MALAT1 on MPM cells.

YAP is a transcriptional co-activator of the Hippo signaling pathway. It is located on human chromosome 11q22. It is currently identified as a candidate oncogene and promotes the gene expression by enhancing the activity of transcription factors [29]. YAP1 is the main splicing isoform of YAP protein. In addition, transcriptional coactivator with PDZ-binding motif (TAZ) is homologous to YAP and has similar functions. When the upstream signal molecule activates MST1/2, the activated MST1/2 binds to SAV1, phosphorylates LATS1/2, and MOB, and the activated LATS1/2 directly phosphorylates YAP/TAZ, binding it to 14-3-3 to stagnate in the cytoplasm [30]. If the pathway is blocked or inactivated, unphosphorylated YAP/TAZ may enter the nucleus from the cytoplasm, and combine with TEADs to promote gene transcription, induce cell transformation, and enhance the cell proliferation, invasion, and metastasis [31]. The abnormal regulation of Hippo signaling pathway is associated with the occurrence and development of diverse types of cancer, such as colorectal cancer [32], gastric cancer [33], breast cancer [34], and MPM [35].

We, in the current research, used bioinformatics tools to determine whether YAP1 is a potential target of miR-141-3p. The results of dual-luciferase reporter analysis revealed that miR-141-3p downregulated the expression level of YAP1. We also found that overexpression of miR-141-3p or knockdown of MALAT1 resulted in a significant decrease in the expression level of YAP1. In addition, the results of cell proliferation assay indicated that the downregulation of the YAP1 expression could reduce the proliferation of MPM cells. Therefore, our data suggested that MALAT1 could interact with miR-141-3p to regulate the development of MPM by targeting YAP1.

In summary, high expression of MALAT1 can promote the proliferation, migration, and invasion of MPM cells. More importantly, our data revealed a new MALAT1/miR-141-3p/YAP1 regulatory pathway in MPM cells. Additionally, MALAT1 can be used as a ceRNA to bind miR-141-3p, which may promote the development of MPM by targeting YAP1.

## Abbreviations

ceRNA: competitive endogenous RNA
FBS: fetal bovine serum
GAPDH: glyceraldehyde 3-phosphate dehydrogenase
lncRNA: long non-coding RNA
MALAT1: metastasis-associated lung adenocarcinoma transcript 1
MPM: malignant pleural mesothelioma
miRNAs: microRNAs
RT-qPCR: quantitative reverse transcription polymerase chain reaction
SDS: sodium dodecyl sulfate
shRNA: short hairpin RNA
TEAD1: TEA domain family member 1
YAP1: YES-associated protein 1
